# A recipe for success? Learning from the rapid adoption of improved chickpea varieties in Ethiopia

**DOI:** 10.1080/14735903.2018.1559007

**Published:** 2018-12-20

**Authors:** Simone Verkaart, Kai Mausch, Lieven Claessens, Ken E. Giller

**Affiliations:** a Eastern and Southern Africa Program, International Crops Research Institute for the Semi-Arid Tropics (ICRISAT), Nairobi, Kenya; b Development Economics Group, Wageningen University & Research, Wageningen, Netherlands; c Impact Acceleration Unit, World Agroforestry Centre, Nairobi, Kenya; d Soil Geography and Landscape Group, Wageningen University & Research, Wageningen, Netherlands; e Natural Resource Management Program, International Institute for Tropical Agriculture (IITA), Arusha, Tanzania; f Plant Production Systems Group, Wageningen University & Research, Wageningen, Netherlands

**Keywords:** Successful adoption, improved chickpea, Ethiopia, panel data, fixed effects

## Abstract

Many studies detail constraints deemed responsible for the limited adoption of new technologies among smallholder farmers in sub-Saharan Africa. By contrast, here we study the conditions that led to the remarkably fast spread of improved chickpea varieties in Ethiopia. Within just seven years, the adoption rate rose from 30 to 80% of the farmers. A combination of factors explains the rapid uptake. Their attraction lay in superior returns and disease resistance. Chickpea was already an important crop for rural households in the studied districts, for both cash income and consumption. Good market access and an easy accessibility of extension services advanced the adoption process. Thus, an attractive technology suitable for rural households in a conducive environment enabled adoption. Our findings prompt us to stress the importance of tailoring agricultural innovations to the realities and demands of rural households, and the need to design and deploy interventions on the basis of *ex-ante* knowledge on factors potentially determining their success or failure.

## Introduction

Agricultural development is critical for sustained poverty reduction in sub-Saharan Africa (Dercon, Gilligan, Hoddinott, & Woldehanna, 2009; World Bank, 2007). Activities designed to address the vulnerability of the African rural poor often promote agricultural innovations to increase productivity, efficiency and ultimately income (Parvan, 2011). Yet, the uptake of new technologies by African smallholders has progressed slowly (van Rijn, Bulte, & Adekunle, 2012; Walker & Alwang, 2015). Indeed, the weak adoption of agricultural technologies in sub-Saharan Africa is a well-documented and widely cited reason for a lack of improvement in agricultural productivity (Headey & Jayne, 2014; World Bank, 2015c). At the same time, there is an increasing pressure to demonstrate the impact, success and ‘value for money’ of agricultural research (Sumberg, Thompson, & Woodhouse, 2013). Therefore, the question why agricultural innovations that appear to be beneficial are not widely adopted by smallholders urgently demands an answer (Zilberman, Zhao, & Heiman, 2012).

Smallholder farmers face numerous barriers and constraints that help to explain the limited adoption of new technologies in sub-Saharan Africa (Woittiez, Descheemaeker, & Giller, 2015). According to the seminal study of Feder, Just, and Zilberman (1985), (under-) adoption is often explained by farm size (Headey & Jayne, 2014; Josephson, Ricker-Gilbert, & Florax, 2014), risk preferences (Dercon & Christiaensen, 2011; Wossen, Berger, & Di Falco, 2015), human capital (Liu & Yamauchi, 2014), labour availability (Jayne, Chamberlin, & Headey, 2014; Ndlovu, Mazvimavi, An, & Murendo, 2014), credit constraints (Holden & Lunduka, 2014), land tenure systems (Beekman & Bulte, 2012; Jin & Jayne, 2013; Melesse & Bulte, 2015), access to input and output markets (Jack, 2011; Jayne, Mather, & Mghenyi, 2010), or by a combination of these (Wakeyo & Gardebroek, 2013). However, there is a lack of understanding of how technological change in smallholder African agriculture actually takes place (Glover, Sumberg, & Andersson, 2016). The large diversity within and among smallholder farming systems affects the uptake of innovations (Franke, van den Brand, & Giller, 2014; Giller et al., 2011). For crops to be adopted and have an impact, they should be equal or superior to conventional varieties (De Groote et al., 2016). The paucity of studies that document the net returns to promising technologies constitutes a surprising gap in the literature (Foster & Rosenzweig, 2010). Sumberg (2005) rightly criticizes agricultural researchers for suggesting too easily that their innovations are not adopted because of well-known constraints, and thus for neglecting the responsibility to contribute to the development process.

Instead of focusing on the lack of adoption of innovations, we studied a contrasting case: that of a dramatic increase in the adoption of improved chickpea varieties in Ethiopia. In just seven years, the percentage of households growing the new varieties rose from 30 to 80%. Improved chickpea varieties are assumed to be a key pro-poor and environmentally friendly technology (Kassie et al., 2009). Grain legumes such as chickpea are both cash and food crops, providing key components of a healthy diet, including proteins and minerals, while helping to reduce the pest and disease build-up associated with cereal mono-cropping and enhancing nitrogen availability for subsequent crops (Franke et al., 2014). However, in order to achieve wide adoption, environmentally sustainable technologies need to generate economic benefits as well (Lee, 2005). Using three rounds of panel data, we have sought to understand what drove the rapid adoption of improved chickpea in Ethiopia. In order to answer this main research question, we formulated three sub-questions:

1. What is the extent of adoption of improved chickpea varieties in the study area?
2. What were the main determinants of improved chickpea adoption?
3. Are economic returns to improved chickpea good predictors of adoption?

Verkaart, Munyua, Mausch, and Michler (2017) used the same data and found that improved chickpea adoption significantly increases household income and reduces household poverty. In this paper, we explore the determinants of adoption that enabled this success. Improved chickpea can be clearly distinguished from local varieties by their seed colour and size. This allowed us to capture adoption accurately and study the mechanisms behind the increase in uptake by farmers with limited misattribution. In addition, we moved beyond dichotomous conceptions of adoption by capturing adoption intensity, dis-adoption, and by assessing the relative importance of chickpea types and varieties, also in relation to other crops, within the farming system. In this way, we wanted to move beyond a narrow focus on adoption constraints in order to study the process leading to wider uptake.

## Background: chickpea in Ethiopia

Ethiopia faces big challenges in agricultural development (Dercon, Hoddinott, & Woldehanna, 2012; Spielman, Byerlee, Alemu, & Kelemework, 2010). It is among the poorest countries in the world, highly drought-prone, and its agricultural sector accounts for 85% of employment. Ethiopia has a population of 92 million that is expected to grow to 160 million by 2050 (Josephson et al., 2014). As a result of population growth, farm sizes have declined rapidly, which has increased the need for agricultural intensification (Headey, Dereje, & Taffesse, 2014). Growth in agriculture is deemed crucial for poverty reduction and food security (Ali, Dercon, & Gautam, 2011). The Ethiopian government has placed agriculture at the centre of its growth strategy (Krishnan & Patnam, 2014), and has declared improved productivity of smallholder agriculture a policy priority (Abebaw & Haile, 2013). Surprisingly, there has been little detailed analysis of the impact of investments in agriculture in Ethiopia (Abro, Alemu, & Hanjra, 2014; Dercon et al., 2009; Spielman et al., 2010).

Chickpea is an important crop in Ethiopia. Ethiopia’s production ranks seventh in the world and accounts for over 90% of chickpea production in sub-Saharan Africa (Kassie et al., 2009; Pachico, 2014). Both seed types of chickpea are grown: (i) Desi varieties that have brown-reddish small seeds, and; (ii) Kabuli types which have cream coloured, larger seeds (Wood, Knights, & Choct, 2011). Despite the fact that Ethiopia’s agro-climatic conditions are suitable to both types, traditionally only Desi chickpea was cultivated. International markets favour the Kabuli types and offer higher prices for them (Shiferaw, Jones, Silim, Teklewold, & Gwata, 2007). This has attracted attention in Ethiopia, and steps have been taken to increase Kabuli production and export (Abera, 2010).

Improvement of productivity and the enhancement of grain quality are essential for the competitiveness of Ethiopia’s chickpea sector, that is, for its ability to provide a consistent supply of the required volumes at competitive prices (Abera, 2010; Keneni et al., 2011). More than ten improved varieties of Desi and Kabuli type chickpea have been released (Asfaw, Shiferaw, Simtowe, & Lipper, 2012). These varieties have various attributes, such as improved yield, better grain quality and disease resistance (Dadi et al., 2005; Keneni et al., 2011). At the beginning of our study period the seed system for Kabuli chickpea production in Ethiopia was in its infancy (Jones, Audi, Shiferaw, & Gwata, 2006). Limited seed access prevented interested farmers from planting improved varieties (Asfaw, Shiferaw, Simtowe, & Haile, 2011). In 2001 less than 1% of the total chickpea area in Ethiopia was covered by improved varieties (Asfaw et al., 2010), which increased to around 18% of farmers in 2003 (Dadi et al., 2005).

In 2004, initiatives were started to accelerate the adoption of improved chickpea varieties in Ethiopia. The Ethiopian Institute of Agricultural Research (EIAR) cultivated partnerships with major actors along the value chain (Abate et al., 2011). Primary co-operatives received breeder seed for multiplication through contracts to enable the dissemination of improved chickpea varieties (Shiferaw et al., 2007). Moreover, the Tropical Legumes II (TLII)^1^ programme supported the establishment of seed grower associations. TLII focused on major chickpea producing areas in the Shewa region for upscaling the cultivation of suitable chickpea varieties and effective marketing strategies (Monyo & Varshney, 2016). Other developments that boosted the chickpea sector were the decision to include chickpea in the Ethiopian Commodity Exchange and the formation of the multi-stakeholder EthioPEA alliance.

## Materials and methods

### Surveys and data

Three districts were selected: Minjar-Shenkora, Gimbichu and Lume-Ejere. They are major chickpea growing areas and have a suitable agro-ecology (Asfaw et al., 2011). The districts are in the Shewa region northeast of Debre Zeit (which lies 50 km southeast of the capital, Addis Ababa). The study area is located in the central highlands at an elevation ranging from 1900–2500 metres above sea level. Debre Zeit Agricultural Research Centre (DZARC) is located in the area and is a source of information and improved varieties (Asfaw et al., 2012).

We utilized three rounds of panel data collected under the TLII project. Farm households were randomly selected; thus non-chickpea growing farmers were also interviewed. During the three survey rounds 700, 661 and 631 households were surveyed in 2006/07, 2009/10 and 2013/14 respectively. We limit our analysis to households that were interviewed in all three rounds of the survey, providing a balanced sample of 606 households with an attrition rate of 13%. To check for non-random attrition we compared characteristics in the 2006/07 season and found no significant differences.

To enable comparisons across time, we deflated nominal Ethiopian Birr values to real values using the national consumer price index with 2005 as a base, following Bezu, Barrett, and Holden (2012). These constant 2005 data were subsequently converted from Ethiopian Birr to US dollars (USD) Purchasing Power Parity (PPP) values, using rates extrapolated from the 2011 International Comparison Program (World Bank, 2015b). Adopters are defined as households who planted an improved chickpea variety in the season surveyed. We account explicitly for input and hired labour costs as well as family labour in our analysis of returns and chickpea productivity.

To add depth to the analysis emerging from the panel data, focus group discussions (FGDs) and semi-structured interviews with experts were conducted in October 2015. Six villages were purposefully selected to reflect differences in market access, low and high adoption rates as well as variations in wealth. A total of seventy-one farmers participated in the FGDs.

### Analysis

Glover et al. (2016) call to move beyond a ‘black box’ conception of adoption as a dichotomous linear process whereby inferior existing material is replaced by a discrete new technology. When farmers opt for innovations such as the introduction of a new variety, they make a decision regarding the intensity of adoption (Marra, Pannell, & Ghadimb, 2003; Sumberg, 2016). It is therefore important to consider how much land is allocated to new varieties compared to other (local) varieties and other crops. We assess various indicators of adoption of the various improved chickpea varieties and types (first sub-question). Specifically, we analyse the share of households as well as land (in hectares and percentage) allocated to improved and local chickpea varieties and to other crops. We also provide information on the kinds of improved chickpea varieties that were adopted and on their characteristics.

We assess the determinants of technology adoption (second sub-question) by comparing descriptive statistics related to the technology, household characteristics and the context of adopters and non-adopters. We assess differences in returns to improved and local chickpeas and compare the yields, costs, labour requirements and prices of improved and local chickpeas to those of other major cereals and legumes, using analysis of variance (ANOVA). We also compare demographics, income, poverty, asset ownership and livelihoods and contextual characteristics, such as market and extension access, rainfall, elevation and soil type. Where we do not have data, we supplement results with findings from the FGDs and from literature. Finally, we assess the value of improved chickpea as a determinant for adoption on the basis of an in-depth analysis of the returns (third sub-question).

Because improved chickpea varieties have not been distributed randomly, adopters and non-adopters may differ systematically (Asfaw et al., 2011). This raises concerns of selection bias where better-skilled farmers or those targeted by technology transfer may be more likely to adopt (Dercon et al., 2009). Indeed, Smale and Mason (2014) found that adopters are generally wealthier in terms of capital and asset endowments and have better access to information, financial services, markets and infrastructure. Therefore, the decision to grow improved varieties is potentially endogenous to household welfare. An advantage of panel data over cross-sectional data is that observed and unobserved time-invariant household characteristics can be separated (Dercon et al., 2009). We utilize fixed effects estimation and further control for time-invariant unobservables by including village time interactions. We focus on the adoption decision here; for a rigorous assessment of the impact of the decision to adopt improved chickpea on income and poverty we refer to Verkaart et al. (2017), where instrumental variable fixed effect models have been applied to the same dataset. We included various covariates in our chickpea yield and gross returns estimations, in order to control for input costs including family labour. Disaggregated results are presented for Kabuli, improved Desi and local Desi types and for specific chickpea varieties.

## Results and discussion

### Adoption of improved chickpea varieties

First, we address the question: *What is the extent of adoption of improved chickpea varieties in the study area?* Improved chickpea varieties became available only relatively recently in the study area. In the 2006/07 season a little more than 30% of the farmers grew improved chickpea varieties while over half of them produced local Desi varieties (Table 1). By 2013/14 the adoption of improved chickpea increased dramatically to 79% of households, representing almost 19% of the total cultivated area and 85% of the chickpea area. In addition, more farmers started cultivating chickpea, with 90% of chickpea growers adopting improved varieties in the 2013/14 season. In terms of the number of growers and the allocated area, chickpea was the third most important crop and the most important legume. Varieties adopted were mainly of the Kabuli type; they particularly substituted the local Desi varieties and, to some extent, wheat and other legumes such as grass pea (*Lathyrus sativus* L.) and field pea (*Pisum sativum* L.). Only 5% of farmers adopted improved Desi varieties. Improved Kabuli varieties were most often adopted by former Desi growers; but also farmers that had not previously grown chickpea adopted them.

**Table 1.** chickpea adoption and planting of other crops.

	Planting of crops (%)			Land allocation (ha)			Land allocation (%)		
Crop	06/07	09/10	13/14	06/07	09/10	13/14	06/07	09/10	13/14
Improved chickpea	31.2	63.0	79.0	0.17	0.33	0.42	5.9	12.1	18.9
Improved Kabuli	30.5	56.9	73.4	0.17	0.30	0.40	5.6	10.9	17.6
Improved Desi	2.0	7.3	5.6	0.01	0.03	0.03	0.3	1.2	1.3
Local Desi	52.8	47.9	25.7	0.22	0.15	0.09	8.9	5.9	3.4
Chickpea	65.5	80.5	88.1	0.39	0.48	0.51	14.8	18.0	22.4
Teff	90.9	94.9	97.2	0.73	0.74	0.74	31.5	29.2	33.4
Wheat	94.7	95.2	93.2	0.80	0.86	0.63	35.0	34.2	28.4
Barley	39.8	37.3	32.1	0.09	0.08	0.07	3.9	3.4	3.0
Maize	26.4	15.8	8.4	0.03	0.04	0.01	1.4	1.2	0.6
Sorghum	4.3	2.5	0.3	0.02	0.02	0.00	1.0	0.5	0.0
Lentil	35.8	53.0	44.5	0.06	0.08	0.07	5.5	7.3	6.7
Faba bean	33.7	39.3	36.4	0.14	0.20	0.17	2.7	3.4	3.3
Grass pea	21.8	19.5	10.3	0.06	0.04	0.02	2.6	1.8	0.9
Field pea	18.2	13.2	10.8	0.04	0.03	0.03	1.6	1.0	1.0
Observations	606			606			606		

Among the improved Kabuli varieties, Arerti was the most popular, followed by Shasho and (initially) Ejere (Table 2). Improved Desi varieties released in the late 70s and early 80s and the more recently introduced Kabuli varieties Chefe and Habru were adopted only by very few farmers. The varieties Arerti and Shasho have the greatest yield potential and tolerance to *Fusarium* wilt. A clear advantage of Arerti is its additional tolerance to *Ascochyta* blight. Both diseases constitute major problems for chickpea production in Ethiopia (Abate, 2012). During FGDs farmers indicated that they preferred Arerti because of its tolerance to these fungal diseases.

**Table 2.** Chickpea variety information.

Chickpea variety	Households planted (%)			Type	Year of release	Yield (t/ha)	Seed size (mm)	Maturity (days)	Tolerance / special trait	Origin
	06/07	09/10	13/14							
Arerti	7.8	19.3	50.3	Kabuli	1999	1.6–5.2	6	105–155	Ascochyta blight, Fusarium wilt	ICARDA
Shasho	14.5	38.6	22.9	Kabuli	1999	1.6–4.6	6–7	90–155	Fusarium wilt	ICRISAT
Ejere	12.7	0.2	0.0	Kabuli	2005	unknown	8–9	unknown	Ascochyta blight, drought	ICARDA
Dubi	0.7	5.8	5.3	Desi	1978	1.7–2.8	5–6	110–115	Bold seed size	DZARC
Habru	0.0	2.8	5.5	Kabuli	2004	unknown	unknown	91–140	Ascochyta blight, drought	ICARDA
Chefe	1.0	0.8	0.3	Kabuli	2004	1.2–4.8	6	95–150	Fusarium wilt, Short duration	ICRISAT
Marye	0.2	0.3	0.3	Desi	1986	1.8–3.0	5–6	106–120	Moisture stress	ICRISAT

Note: Debre Zeit Agricultural Research Center (DZARC), International Center for Agricultural Research in the Dry Area (ICARDA); International Crops Research Institute for the Semi-Arid Tropics (ICRISAT).

**Figure 1.**
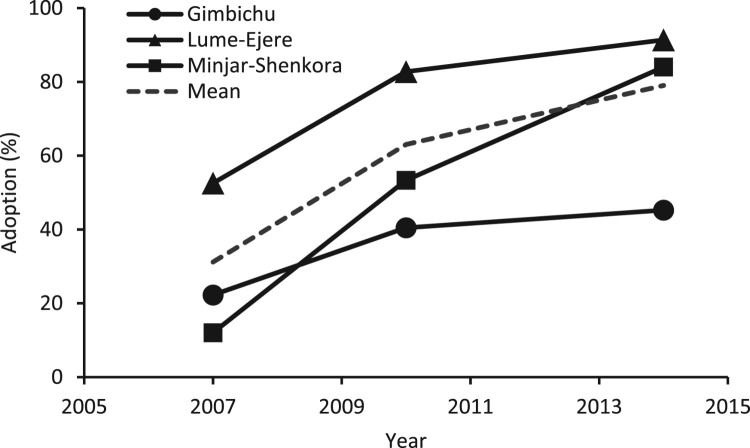
Adoption of improved chickpea by season and district.

A majority of the farmers in Lume-Ejere were already growing improved chickpea in 2006/2007 (52%) and by 2013/2014 almost all households (91%) had adopted the new varieties (Figure 1). In Minjar-Shenkora, only a few households grew improved chickpea varieties (12%) in the 2006/07 season, but by the end of the study the majority of farmers (84%) had adopted them. Gimbichu had some initial adopters (22%), but saw a relatively limited increase in adoption to less than half of the farmers (45%).

### Determinants of adoption

In this section we address the second sub-question: *What were the main determinants of improved chickpea adoption?* Agricultural technologies can be defined as discrete inputs – either goods or methods – which serve to control and manage animal or vegetative growth (Parvan, 2011). Adoption decisions are influenced by many factors (Anderson & Feder, 2007). These factors can be broadly divided into characteristics of the technology, of the users and of the context within which adoption takes place (Biagini, Kuhl, Gallagher, & Ortiz, 2014). We consider each of these in turn.

#### Technology characteristics

The promotion of Kabuli varieties began in 2004. Kabuli types are clearly distinguishable from the Desi type due to their different grain size and colour, which may have had a positive effect on uptake by facilitating trialability, observability and learning (Rogers, 1962). These characteristics make it easier to learn about a new technology and its returns in settings where it is introduced (Foster & Rosenzweig, 2010). Access to improved seeds is another pre-condition for adoption (Asfaw et al., 2012). Improved varieties that had been multiplied by contract farmers were introduced via revolving seed funds, whereby a farmer pays in kind with seed after harvest, and through seed grower associations (Monyo & Varshney, 2016). Finally, as chickpea is a self-pollinating crop, the improved varieties could spread from farmer to farmer (Gwata, 2010).

Of course, the technology needs to be attractive in order to be adopted. We compared local and improved chickpea yields, returns and sales data for growers and sellers (Table 3). Improved chickpea yielded >20% more grain than local varieties; increases in net returns ranged from 50 to more than 200%. The larger land and initial labour allocations as well as increased input and hired labour costs called for by improved chickpea cultivation were easily compensated for by higher prices and productivity. Larger yields could be related to the higher labour and input use, but also to the enhanced yield potential and disease resistance of the improved varieties (Dadi et al., 2005; Keneni et al., 2011). The Kabuli varieties fetched considerably higher prices than Desi varieties due to a growing demand in both domestic and export markets (Abera, 2010; Shiferaw & Teklewold, 2007). Although more farmers sold local Desi types in the first round, the relation was reversed 2013/14, with 46% of growers selling local Desi and 83% selling improved chickpea. During the focus group discussions, farmers indicated that the market demand for Desi was largely replaced by Kabuli. Consequently, improved varieties provided an important source of cash, contributing 35–45% of the total crop sales income, compared with 18–22% for local Desi.

**Table 3.** Production, costs and returns of improved and local chickpeas.

	2006/07			2009/10			2013/14		
	Local	Improved	t-test	Local	Improved	t-test	Local	Improved	t-test
Productivity (kg/ha)	1,917	2,315	***	1,998	2,377	***	1,933	2,472	***
Returns to land (USD/ha)	2,517	3,785	***	1,704	3,679	***	1,165	2,016	***
Cultivated area (ha)	0.42	0.55	***	0.32	0.52	***	0.33	0.54	***
Family labour (days/ha)	75.5	85.0	**	82.8	73.5	*	75.6	74.4	
Crop cost (USD/ha)	277	529	***	234	424	***	234	338	***
Sold crop (yes = 1, no = 0)	0.87	0.80	**	0.67	0.86	***	0.46	0.83	***
Producers (Obs.)	320	197		290	388		156	479	
Sales price (USD)	1.45	1.90	***	0.97	1.72	***	0.76	0.95	***
Production sold (%)	58.3	71.1	***	51.3	60.4	***	35.6	56.4	***
Share of crop sales income (%)	22.2	35.3	***	18.0	44.5	***	22.1	41.1	***
Sellers (Obs.)	280	156		193	332		72	398	

Note: Significance levels *p < 0.10, **p < 0.05, ***p < 0.01.

Adoption generally implies a reallocation of resources (Bevan, Collier, & Gunning, 1990). The decision which crops to plant thus depends, at least partially, on a weighing of the investments (capital, land and labour) against the expected returns (Table 4). Kabuli generated the third largest returns among crops, outperformed only by lentil and wheat (in the 2013/14 season). While the cultivation of improved Kabuli incurred more costs than the growing of the other legumes in the first two survey rounds, it was less costly than cereals. This is to be expected, as legumes require smaller amounts of fertilizer. Although the seed rate for chickpea is large compared with cereals (Kassie et al., 2009), farmers can save seed (Asfaw et al., 2010). Furthermore, the capacity of legumes to fix atmospheric nitrogen can reduce the need for chemical fertilizer use and bring down the costs of subsequent cereal production (Giller, 2001). The economic benefits of enhanced cereal production and reduced fertilizer costs are not taken into account in our analysis. Chickpea was highly marketable with around 80% of households selling improved Kabuli and Desi. Chickpea fetched better prices than most crops, with the exception of teff and lentil in the last two rounds. There were no pronounced differences in terms of family labour allocation.

**Table 4.** Comparison of chickpea and other crop production characteristics (of growers / sellers).

		Improved Kabuli	Improved Desi	Local Desi	Teff	Wheat	Faba bean	Lentil
Productivity (kg/ha)	2006/07	2,342_a_	2,167_a,b,d_	1,917_b_	1,568_c,e_	2,506_a_	1,650_c,d_	1,438_e_
	2009/10	2,455_a_	1,757_b,d,f_	1,998_b,c_	1,549_d_	2,740_e_	1,866_c,f_	1,498_d,g_
	2013/14	2,477_a_	2,414_a_	1,933_b_	1,738_c_	2,786_d_	1,988_b_	1,503_e_
Return to land (USD/ha)	2006/07	3,828_a_	3,337_a,b_	2,517_b_	1,956_c_	2,231_d_	1,825_c_	2,518_b,e_
	2009/10	3,804_a_	2,658_b_	1,704_c,e_	2,066_d_	1,838_c_	1,463_e_	2,563_b_
	2013/14	2,006_a_	2,145_a,c,e_	1,165_b_	1,847_c_	1,567_d_	1,273_b_	2,218_e_
Family labour (days/ha)	2006/07	86_a_	63_a,b,c_	75_b,c_	82_a,b_	71_c_	89_a_	89_a_
	2009/10	74_a_	72_a,b_	83_b,c_	89_c,d_	68_a_	93_d_	85_b,c,d,e_
	2013/14	74_a_	86_a_	76_a_	85_a_	82_a_	87_a_	90_a_
Crop cost (USD/ha)	2006/07	536_a_	507_a,b,c_	277_b_	739_c_	827_d_	325_b,e_	297_b,f_
	2009/10	433_a_	342_a,e,f_	234_b_	684_c_	777_d_	250_b,e,f_	302_f_
	2013/14	341_a_	305_a,b_	234_b_	714_c_	808_d_	260_b,e_	337_a_
Sold crop (yes = 1, no = 0)	2006/07	0.79_a_	0.75_a,b_	0.87_b_	0.80_a_	0.83_a,b_	0.45_c_	0.84_a,b_
	2009/10	0.85_a_	0.86_a,d_	0.67_b_	0.66_b_	0.63_b_	0.47_c_	0.79_d_
	2013/14	0.83_a_	0.85_a,c_	0.46_b,d_	0.72_c_	0.53_b_	0.44_d_	0.73_c,e_
Sales price (USD)	2006/07	1.91_a,f_	1.68_a,b,c_	1.45_b_	1.76_c_	1.23_d_	1.34_e_	1.99_f_
	2009/10	1.72_a_	1.68_a_	0.97_b_	1.88_c_	0.98_b_	0.94_b_	1.88_c_
	2013/14	0.95_a_	0.97_a_	0.76_b_	1.50_c_	0.87_d_	0.77_b_	1.69_e_

Note: Values in the same row and subtable not sharing the same subscript are significantly different at *p *< .05 in the two-sided test of equality for column means. Cells with no subscript are not included in the test. Tests assume equal variances.

#### Household characteristics

We found systematic differences between adopters and non-adopters in demographics, welfare and livelihood indicators (Table 5). Adopters had larger households in the first two rounds and lower dependency rates in the last round. They more often hired labour in the first two rounds. Initial adopters were also slightly better-educated, though overall education levels were low.

**Table 5.** Comparison of adopter and non-adopter household characteristics.

	2006/07			2009/10			2013/14		
	Non-adopter	Adopter	t-test	Non-adopter	Adopter	t-test	Non-adopter	Adopter	t-test
*Demographics*									
Household size (No.)	6.08	6.76	***	6.00	6.59	***	5.63	5.81	
Dependents (%)	42.9	45.4		39.0	40.9		39.9	34.9	**
Hired labour (yes = 1, no = 0)	0.57	0.78	***	0.59	0.70	***	0.57	0.63	
Male head (yes = 1, no = 0)	0.93	0.96		0.94	0.95		0.91	0.91	
Education head (years)	1.59	1.98	*	1.87	1.99		2.14	1.81	
Age head (years)	46.3	47.9		49.3	48.1		50.3	52.0	
*Income and poverty*									
Total net income (USD)	4,541	7,760	***	4,145	7,008	***	3,404	4,696	***
Income per capita (USD)	837	1,232	***	806	1,175	***	670	885	***
Poor household (< $1.25)	0.28	0.11	***	0.37	0.20	***	0.48	0.27	***
Poor household (< $2.00)	0.57	0.32	***	0.58	0.39	***	0.70	0.54	***
*Assets and livelihood*									
Value assets (USD)	363	477	**	325	376	*	493	722	***
Land owned (ha)	2.01	2.67	***	2.00	2.41	***	1.94	2.17	*
Livestock owned (TLU)	4.77	7.33	***	4.91	6.23	***	4.58	5.04	
Off-farm income (yes = 1, no = 0)	0.29	0.24		0.30	0.21	**	0.39	0.25	***
Crop share total income (%)	89.8	91.5		85.3	90.7	***	80.8	87.8	***
Observations	417	189		224	382		127	479	

Note: Significance levels *p < 0.10, **p < 0.05, ***p < 0.01.

Adopters were wealthier, having consistently greater incomes. Even though nominal incomes increased considerably, real incomes could not keep up with the high inflation. In 2011, for example, Ethiopian food price inflation was 39%, three times the sub-Saharan African average of 13% (World Bank, 2015a). As a result, the real incomes of both adopters and non-adopters shrank during the study period. Despite this loss in real per capita income, most households remained above the US$1.25 poverty threshold.

Adopters owned more assets, land and livestock, though the differences became smaller over time as more households moved into the adopter category. Households owned on average more than 2 ha of land, which makes their farm sizes relatively large, considering that 80% of the farms in sub-Saharan Africa are now smaller than this (Lowder, Skoet, & Singh, 2014). Regarding livelihood diversification, non-adopters participated more in off-farm income-generating activities and therefore had lower crop income shares in the last two rounds. Still, the effect of livelihood diversification was limited, as crop income contributed 80–90% of the total income.

Rogers (1962) indicated that technologies need to be compatible with the existing preferences, needs and practices of adopters. Examples include taste preferences as well as specific processing and storage requirements (Lunduka, Fisher, & Snapp, 2012). In terms of taste preferences, the focus group discussions revealed that farmers adjusted well to the newly introduced Kabuli varieties. Furthermore, Kabuli varieties were said to be easier to process due to their thinner seed coats which countered issues around poorer storability. Hence, it is likely that in this case taste and other preferences were facilitating the adoption process, rather than hindering it.

#### Context

Adoption choices are conditioned by the context – comprising, among other things, access to markets and extension services, agro-ecological conditions, and land tenure systems. Development actors need to take the specific context into account when designing interventions (Oumer, Hjortsø, & de Neergaard, 2013).

The functionality and structure of value chains and the access to markets affect input and output prices and transportation costs (Chamberlin & Jayne, 2013). The three selected districts are adjoining, and differences in market access are relatively small (Table 6). The sites are close to Addis Ababa and other major markets, as Debre Zeit and Adama, and roads in the area are generally good. The FGDs revealed that market information, notably on prices, was available and known to farmers. Despite good overall market access, adopters tended to be more numerous in areas closer to main markets.

**Table 6.** Comparison of adopter and non-adopter context characteristics.

	2006/07			2009/10			2013/14		
	Non-adopter	Adopter	t-test	Non-adopter	Adopter	t-test	Non-adopter	Adopter	t-test
Travel time to main market (min)	210	167	***	218	184	**	248	183	**
Extension contact (yes = 1, no = 0)	0.87	0.94	**	0.94	0.97	**	0.94	0.98	**
Extension contacts (days/year)	5.0	6.9	***	11.5	13.6	**	16.7	17.0	
Average rainfall past 5 seasons (mm)	595	605	**	636	614	***	632	590	***
St. dev. rainfall past 5 seasons (mm)	95.6	102.3	***	54.9	59.6	***	70.9	83.9	***
Elevation (m above sea level)	2,073	2,136	***	2,134	2,069	***	2,269	2,046	***
Black soil (yes = 1, no = 0)	0.96	0.98		0.95	0.98	*	0.97	0.97	
Sandy soil (yes = 1, no = 0)	0.81	0.71	***	0.83	0.75	**	0.81	0.77	
Mixed soil (yes = 1, no = 0)	0.25	0.23		0.27	0.23		0.23	0.25	
Observations	417	189		224	382		127	479	

The adequate and timely access to relevant advice and training can influence adoption (Anderson & Feder, 2007). Indeed, adopters had better access to extension services, though extension access was almost universal and contacts were quite frequent across both adopters and non-adopters. This reflects Ethiopia’s intensive public extension system (Gebremedhin, Jaleta, & Hoekstra, 2009; Krishnan & Patnam, 2014), which has an extension-worker-to-farmer ratio of 1:476. This ratio is 1:1000 for Kenya, 1:1603 for Malawi and 1:2500 for Tanzania (Abate et al., 2015). The higher intensity of extension contacts of (early) adopters, suggests that extension had a positive effect on uptake. The high share of initial adopters in Lume-Ejere district also supports this assertion, as Asfaw et al. (2012) noted that the district benefited from pre-extension demonstrations and improved seed distribution trials, which gave the local farmers a head start in the adoption process. While farmer-to-farmer technology transfer is generally important, the initial adoption was clearly facilitated by a strong extension system allowing more innovative farmers to try the technology.

Agro-ecological characteristics, such as soil quality, the rainfall amount and the distribution and farming systems, can be important variables determining differences in adoption (Feder & Umali, 1993; Mason & Smale, 2013). Although variations in climate are relatively minor in the study area, it is located along a gradient: from higher elevation and precipitation in Gimbichu (2411 metres, 675 mm) to lower elevation and precipitation in Minjar-Shenkora (1896 metres, 565 mm). Initial adoption rates were highest in the central district of Lume-Ejere (50%). Minjar-Shenkora soon caught up with Lume-Ejere. The high-elevation, high-rainfall area of Gimbichu had the lowest adoption rates (45%); farmers there continued the cultivation of local Desi varieties (71%) and lentil (74%). The data and FGDs reveal that the agro-climatic conditions in Gimbichu were less favourable for chickpea cultivation, particularly because of the higher rainfall in combination with vertisols which are prone to waterlogging. Chickpea is highly sensitive to waterlogging and is grown largely with residual moisture (Agegnehu & Sinebo, 2011). Because improved Kabuli varieties take approximately two weeks longer to mature than local Desi varieties, their cultivation in Gimbichu required relatively labour-intensive practices to remove excess moisture. Due to their shorter duration this is not required for local Desi and lentil, which may explain the weaker adoption of the new varieties in Gimbichu.

Tenure security (Melesse & Bulte, 2015) and access to credit (Foster & Rosenzweig, 2010) are also potential determinants of or obstacles to adoption. As we did not collect detailed data on this, we rely on the FGDs to assess their influence on adoption. As in the rest of Ethiopia, land is state-owned with individuals given usufruct rights. This means that land cannot be sold, permanently exchanged for other property or mortgaged; and it can only be inherited by the immediate family (Ali et al., 2011). Though chickpea is an annual crop and requires less long-term investments than perennials, its residual soil fertility benefits, including increased yields of subsequent cereal crops, are part of its appeal to farmers (Giller, 2001). Recent land certification provided incentives for farmers to invest in their land (Wakeyo & Gardebroek, 2013). However, it seems that the lack of property rights did not negatively influence the adoption of improved chickpea. There is widespread availability of credit in Ethiopia, particularly for inputs (Dercon & Christiaensen, 2011; Krishnan & Patnam, 2014). Data collected in the first round indicated that over 80% of households had access to credit (Asfaw et al., 2012); this makes it unlikely that credit was a constraint for uptake.

### Returns to improved chickpea

In this section we address the question: *Are economic returns to improved chickpea good predictors of adoption?* Profits or net returns fluctuate with output and price levels and with changes in expenses related to input adjustment (de Janvry, Dunstan, & Sadoulet, 2011). Labour and capital investments associated with adoption thus need to be considered (Jack, 2011). Using fixed effects (FE) estimation, we assessed the effect of improved chickpea adoption on yields and returns (Tables 7 and 8).

**Table 7.** Fixed Effects (FE) estimation. Dependent variable: Ln chickpea yield (kg/ha).

	(1)	(2)	(3)	(4)	(5)
VARIABLES	FE	FE	FE	FE	FE
Improved chickpea (yes = 1, no = 0)	0.1126 (0.106)				
Ln improved chickpea seed (kg)		0.0143 (0.020)			
Improved chickpea (% chickpea area)			0.0010 (0.001)		
Kabuli (yes = 1, no = 0)				0.0808 (0.098)	
Improved Desi (yes = 1, no = 0)				0.1483 (0.158)	
Local Desi (yes = 1, no = 0)				−0.0268 (0.067)	
Arerti (yes = 1, no = 0)					0.1362 (0.083)
Shasho (yes = 1, no = 0)					0.1046* (0.061)
Ejere (yes = 1, no = 0)					−0.0814 (0.103)
Dubi (yes = 1, no = 0)					0.1780 (0.196)
Habru (yes = 1, no = 0)					−0.1304 (0.105)
Chefe (yes = 1, no = 0)					0.3309* (0.171)
Marye (yes = 1, no = 0)					0.2605 (0.284)
Constant	3.9610 (4.170)	3.9159 (4.164)	3.8282 (4.156)	3.9127 (4.178)	3.4680 (4.203)
Observations	1,419	1,419	1,419	1,419	1,419
Households	581	581	581	581	581
Rho	0.512	0.510	0.510	0.512	0.518
R-squared overall	0.125	0.125	0.126	0.124	0.122

Note: Columns present fixed effects regressions for various indicators of improved chickpea adoption. Regressions include time-varying explanatory variables indicated in the Annex, household fixed effects, year dummies and village time interactions. Fully robust standard errors in parentheses (*p < 0.10, **p < 0.05, ***p < 0.01).

**Table 8.** Fixed Effects (FE) estimation. Dependent variable: Ln gross chickpea return (USD/ha).

	(1)	(2)	(3)	(4)	(5)
VARIABLES	FE	FE	FE	FE	FE
Improved chickpea (yes = 1, no = 0)	0.3865*** (0.109)				
Ln improved chickpea seed (kg)		0.0698*** (0.020)			
Improved chickpea (% chickpea area)			0.0047*** (0.001)		
Kabuli (yes = 1, no = 0)				0.2870*** (0.101)	
Improved Desi (yes = 1, no = 0)				0.3197** (0.161)	
Local Desi (yes = 1, no = 0)				−0.1510** (0.067)	
Arerti (yes = 1, no = 0)					0.2934*** (0.085)
Shasho (yes = 1, no = 0)					0.2920*** (0.064)
Ejere (yes = 1, no = 0)					0.0459 (0.106)
Dubi (yes = 1, no = 0)					0.3873* (0.198)
Habru (yes = 1, no = 0)					−0.0192 (0.103)
Chefe (yes = 1, no = 0)					0.5778*** (0.180)
Marye (yes = 1, no = 0)					0.3262 (0.342)
Constant	8.3921** (4.184)	8.2628** (4.188)	7.8409* (4.160)	8.5421** (4.187)	7.6196* (4.221)
Observations	1,419	1,419	1,419	1,419	1,419
Households	581	581	581	581	581
Rho	0.536	0.530	0.527	0.536	0.533
R-squared overall	0.138	0.141	0.151	0.137	0.139

Note: Columns present fixed effects regressions for various indicators of improved chickpea adoption. Regressions include time-varying explanatory variables indicated in the Annex, household fixed effects, year dummies and village time interactions. Fully robust standard errors in parentheses (*p < 0.10, **p < 0.05, ***p < 0.01).

Despite promising on-station results (Table 2), we observed no significant increase in yield due to the adoption of improved varieties. However, Chefe and Shasho did have between 10 and 33% higher yields (albeit only significant at *P *< 0.1). It seems other chickpea varieties performed less well. Another possible explanation is that the yield difference occurs mainly from the disease resistance and therefore only shows during seasons of high pressure. In fact, FGDs suggested that disease resistance was an important incentive for adoption. There were significant, consistent, strong positive effects of improved chickpea adoption on chickpea returns, with 38% higher returns to improved chickpea. Moreover, using the same dataset, Verkaart et al. (2017) found that improved chickpea adoption significantly increased household income, while reducing household poverty. When disaggregating the analysis by chickpea type, it becomes clear that the results are related to both Kabuli and Desi adoption. Further disaggregating results by variety shows that returns to Chefe, Dubi (Desi), Arerti and Shaso (from high to low) were significant, with 29 and 57% higher returns. This suggests that net returns are an important predictor of adoption and emphasizes the need to carefully measure benefits and costs associated with new technologies in order to explain adoption decisions.

## Conclusions

We studied a case of successful adoption of improved chickpea varieties in Ethiopia using panel data, and tried to explain the success. We looked at yields and returns. The results for yields were unclear. Improved chickpea cultivation did, however, result in higher returns, largely due to the higher prices for the new varieties. Our analysis suggests that innovative technologies are more readily adopted if they offer distinct and measurable benefits to facilitate adoption: such as high returns, disease tolerance or, as in our case, both. Other determinants that positively influenced adoption were the traditional importance of chickpea for livelihoods and within the farming system, as well as the good accessibility of markets and extension services. Overall, it seems that the rapid adoption of the new varieties of chickpea was enabled by three main factors: the new technology was visibly distinct, and perceived as attractive; it was considered to be suitable for local households, and the environment was conducive to its introduction.

Noteworthy in our case is the absence of almost any negative trade-offs: the technology was not overly complex or demanding in terms of labour, inputs or cash investment. Someone might wonder whether all aspects always have to be right for adoption to take place. We would like to reverse the question and ask: Why promote a technology when it increases risks or entails costs without sufficient rewards? When it cannot be adopted due to various constraints and market imperfections? When it is too complex for the target group to understand? When households do not have sufficient land or are diversifying away from agriculture? When it does not fit taste preferences, or when the agro-climatic conditions are not conducive to its adoption? Success in technology adoption may not depend on getting everything right, but on getting some important things right and avoiding many different causes of failure.

People will only adopt a new technology if they expect benefits from it. As adoption involves risks, learning and investments, these benefits need to be substantial, particularly in the case of resource-poor smallholders. In the end, only innovations that clearly outperform locally available technologies and manifest limited downside risks are likely to be adopted on a large scale. Though our results suggest that returns are good predictors of adoption, those returns are influenced by many external factors beyond the control of technology transfer interventions. A good understanding of the local context and the attractiveness of a technology for a diversity of households, can provide information about potential benefits and pitfalls to avoid. This emphasizes the importance of careful site selection and targeting when disseminating innovations to ensure successful uptake. Robust evidence on what works, where and why, can be vastly instrumental in effectively assisting poor farmers. Thus, if we want to design and deploy more successful interventions, agricultural research for development efforts need to more carefully consider the realities of smallholders.

## Note

1 Tropical Legumes II was funded by the Bill & Melinda Gates Foundation to enhance grain legume productivity and production to increase poor farmers’ income in drought-prone areas of sub-Saharan Africa and South Asia. For more information see http://www.icrisat.org/TropicalLegumesII/.

## Annex

**Table A1.** Explanatory variable descriptives.

	2006/07		2009/10		2013/14	
Variables	Mean	SD	Mean	SD	Mean	SD
Chickpea yield (kg/ha)	2,040	1,100	2,192	1,132	2,374	1,080
Gross chickpea return (USD/ha)	3,276	1,807	3,261	1,961	2,193	1,053
Improved variety (1 = yes, 0 = no)	0.312	0.464	0.630	0.483	0.790	0.407
Improved chickpea seed (kg)	34.23	79.27	60.70	80.05	89.60	101.8
Improved chickpea (% chickpea area)	22.63	37.08	51.10	43.43	72.74	40.58
Kabuli (yes = 1, no = 0)	0.305	0.461	0.569	0.496	0.734	0.442
Improved Desi (yes = 1, no = 0)	0.020	0.139	0.073	0.260	0.056	0.230
Local Desi (yes = 1, no = 0)	0.528	0.500	0.479	0.500	0.257	0.438
Arerti (yes = 1, no = 0)	0.078	0.268	0.193	0.395	0.503	0.500
Shasho (yes = 1, no = 0)	0.145	0.353	0.386	0.487	0.229	0.421
Ejere (yes = 1, no = 0)	0.127	0.333	0.002	0.041	0.000	0.000
Dubi (yes = 1, no = 0)	0.007	0.081	0.058	0.233	0.053	0.224
Habru (yes = 1, no = 0)	0.000	0.000	0.028	0.165	0.055	0.227
Chefe (yes = 1, no = 0)	0.010	0.099	0.008	0.091	0.003	0.057
Marye (yes = 1, no = 0)	0.002	0.041	0.003	0.057	0.003	0.057
Male head (1 = yes, 0 = no)	0.936	0.246	0.942	0.233	0.914	0.280
Household size (No.)	6.295	2.250	6.368	2.358	5.772	2.089
Dependents (%)	43.70	20.49	40.21	19.62	35.98	21.60
Off-farm income (1 = yes, 0 = no)	0.276	0.447	0.246	0.431	0.282	0.450
Land owned (ha)	2.215	1.308	2.257	1.299	2.122	1.281
Average rainfall past 5 seasons (mm)	97.70	15.50	57.85	12.64	81.18	12.04
St. dev. rainfall past 5 seasons (mm)	598.0	47.65	622.4	52.93	599.2	50.91
Chickpea seed (USD/ha)	270.9	234.4	272.7	123.6	189.7	98.04
Chickpea fertilizer (USD/ha)	20.98	86.95	12.83	72.47	18.48	78.75
Chickpea own manure (kg/ha)	9.049	67.52	11.40	184.0	27.48	343.2
Chickpea chemicals (USD/ha)	21.43	77.98	27.78	43.13	54.13	78.79
Chickpea hired oxen (USD/ha)	0.277	5.510	1.226	14.00	0.278	6.248
Chickpea hired labour (USD/ha)	33.72	78.64	32.01	75.08	53.07	99.69
Chickpea family labour (days/ha)	75.14	44.03	76.57	69.06	74.47	47.13
Observations	606		606		606	

Note: Chickpea production covariates were transformed to natural logarithm (ln) for the fixed effects estimation.
